# Possible Future Career Challenges and Associated Factors among Dental Students and Interns

**DOI:** 10.1155/2020/9730125

**Published:** 2020-04-09

**Authors:** Sarah Fita, Faris Alshuraim, Abdullah Almulhim, Jehan AlHumaid, Muhanad Alhareky, Muhammad Nazir

**Affiliations:** ^1^Department of Preventive Dental Sciences, College of Dentistry, Imam Abdulrahman Bin Faisal University, P. O. Box 1982, Dammam 31441, Saudi Arabia; ^2^Department of Dental Education, College of Dentistry, Imam Abdulrahman Bin Faisal University, P. O. Box 1982, Dammam 31441, Saudi Arabia

## Abstract

**Background:**

Dental students face a variety of challenges as they enter the dental profession. The study aimed at assessing dental students' opinions about their perceived future career challenges in the dental profession and the associated factors.

**Materials and Methods:**

This cross-sectional study of 637 students representing different public and private dental colleges was conducted in Saudi Arabia. The participants responded to a pretested questionnaire conducted online.

**Results:**

There were 59.7% (*n* = 380) of males and 40.3% (*n* = 257) of females, and the mean age of the students was 21.80 (±2.14) years. The majority of participants thought that they will have difficulties in establishing their private clinic (63.9%) and finding a government job (>60%). Senior students were more likely to perceive getting a government job (OR = 1.79, *P* = 0.02), securing an admission into specialty program (OR = 2.75, *P* = 0.001), and establishing a private dental clinic (OR = 2.51, *P* = 0.001) as future career challenges than junior students. Similarly, female gender was associated with increased perception of challenges about finding a government job (OR = 1.911, *P* = 0.002), getting an admission into specialty program (OR = 1.5, *P* = 0.038), and establishing a private clinic (OR = 2.02, *P* = 0.001). In addition, low academic score and low level of father's education were associated with increased odds of perceiving challenges of finding a government job and getting an admission into specialty programs.

**Conclusions:**

Establishing a private clinic and getting a government job were the most common career challenges. Senior students, female students, and students with low academic scores had increased likelihood of facing employment and academic-related difficulties.

## 1. Introduction

For many years around the world, young students have been considering dentistry as their future career choice mainly for the financial security and ease of employment [1–3]. However, in recent years, few stories were arising in the media for newly graduated dentists in Saudi Arabia unable to secure suitable jobs. These headline stories became a topic of discussion for major TV shows as well as social media outlets [4]. These stories arise in the light of a huge increase in the number of dental schools in Saudi Arabia reaching 27 colleges (19 governmental and 8 private) in 2018 compared to 3 governmental dental schools in 1987 [5, 6]. The number of graduating students (both males and females) from these dental schools reached up to 1,956 in 2018 [5]. Similarly, the number of dentists increased from 786 in 1987 to 12,785 in 2014 in Saudi Arabia [6, 7]. The accelerated increase in the number of healthcare graduates was the reason for the Saudi Commission for Health Specialties (SCHS) to publish a report for the first time to discuss the workforce for healthcare professional in Saudi Arabia in the next 10 years. This report addressed the situation of all healthcare providers and followed by recommendations workshop to address the findings [5].

It is normal for healthcare providers to face different challenges as they enter the workforce [8, 9]. These challenges may arise as part of the transition from a student to a practitioner. Students also begin to perceive certain levels of future career challenges at an undergraduate level [8]. It is critical in the dental field for students to know the challenges associated with their future career as there is a rare chance that a dentist can change his/her career after entering the profession [10, 11]. Concern about one's future career while in dental school appears to be a global issue. For example, in China, only 4.7% of the final-year students were very confident of finding an ideal practice position, 40.9% stated it was not difficult, and more than half of the students (54.4%) considered finding an ideal practice position difficult immediately following graduation. In addition, when they were asked about the most important reasons of facing difficulty in finding a job, lack of employment opportunities (43%) was first followed by lack of work experience (40.9%) and having only an undergraduate degree (26.8%) was ranked third [12]. In the UK, a survey among final-year dental students in London University showed more than half (60%) of the respondents anticipated working full-time and 27% expected working part-time in the long term [13]. In Saudi Arabia, fifth-year male and female students at King Saud University considered postgraduate program a necessity for their career (72.4%), perceived opening up of new dental colleges as a threat to the dental profession in the country (59.3%), and thought that establishing dental practice is time-consuming (62.3%) [14].

In a sample of dental students at the University of Jordan who were asked if they would choose dentistry again as a career, 33.3% of them, mostly in the fifth year, said they would not join dental profession if given second chance. Limited employment opportunities, stress, lack of social interaction, and difficult coursework in dentistry were the reasons for not choosing dental career again [9]. The reasons given by the students indicate their sense of future challenges in the career, but no further questions assessed these issues.

Dental students perceive a multitude of potential career challenges; however, there is limited evidence about dental students' perception regarding the challenges they might face as they enter the profession. Also, the abrupt increase of dental schools in Saudi Arabia and the influx of newly graduating dentists in the market are expected causes of some stress. Therefore, there was a need to conduct a study involving students from both private and public dental colleges across Saudi Arabia at this time. The study aimed at assessing dental students' opinions about their future career challenges in the dental profession. In addition, some factors associated with the perception of career challenges were also investigated.

## 2. Materials and Methods

A cross-sectional study was conducted in seven dental colleges in Saudi Arabia. A total of 637 students representing different private and public dental colleges in Saudi Arabia participated in the study through an online survey, between February 2017 and April 2017. Both male and female students from all years as well as dental interns participated in the study. Based on the available career opportunities and associated potential challenges for graduating dentists, the items of the questionnaires were developed and were discussed several times among researchers. These deliberations ensured the face and content validity of the questionnaire. A five-point Likert scale was used for these items. In addition, the questionnaire inquired about the demographic data from study participants. Internal consistency of the items that evaluate future career challenges was measured and found to be satisfactory (Cronbach alpha = 0.80). Further, the questionnaire was pilot-tested on forty dental students to minimize ambiguity and misinterpretation of questions.

The research proposal was approved by the Scientific Research Unit, College of Dentistry at Imam Abdulrahman Bin Faisal University, Dammam, Saudi Arabia. Students and interns from seven dental colleges representing different regions of the country participated in the study. The permission was obtained from different dental colleges to collect data from their respective institutions. The participants were informed of the purpose and benefits of the study. The confidentiality and privacy of the participants were maintained during the administration of the questionnaire, data analysis, and interpretation of the results. The study was conducted according to the principles of the Helsinki Declaration and conforms to the STROBE statement for observational studies.

Data were entered in Statistical Package for Social Sciences (version 22.0, SPSS Inc., Chicago, IL). Descriptive analysis was performed to calculate frequencies of categorical variables. Multivariate logistic regression was performed to evaluate the association between independent variables of the study such as gender, year of study, academic score, parental income, and education with the perceptions about future career challenges. A *P* value <0.05 was used to determine statistical significance.

## 3. Results

Six hundred and thirty-seven students completed the questionnaire. The mean age of the students was 21.80 (±2.14) years. The sample consisted of 59.7% (*n* = 380) of males and 40.3% (*n* = 257) of females. One-third of students (33.9%) had >90% score in the last academic year. The monthly family income was more than SAR 20,000 in 40% of the respondents. About half of the participants' fathers (48.8%) and 33% of mothers had a university education (Table 1).

Table 2 shows the distribution of students' responses about seven career challenges after graduation. Agree and strongly agree scales of each item were combined to present single (%) frequency of positive responses. In the survey, 63.9% of the participants thought that they will have difficulty in establishing their own private clinic, while only 37.6% thought they will have difficulty in finding a job in a private clinic. Almost half of the participants considered it difficult to secure acceptance in Saudi Dental Board or in a graduate program abroad and to get the position of a demonstrator/teaching assistant in academia. Sixty-two percent of the participants agreed that they would face competition in getting a job in the Ministry of Health, and 60.7% thought they would have difficulty in getting a job in the Armed Forces hospitals. Regarding gender differences, a greater proportion of female students perceived career challenges than male students (Table 2).

Different variables of the study were dichotomized to calculate odds ratios. The challenges of getting a government job, establishing a private dental clinic, and securing an admission in specialty program were dichotomized into agree and disagree. Similarly, fifth-year students, sixth-year students, and interns were categorized as senior students while second-year, third-year, and fourth-year students were categorized as junior students.  Table 3 shows both adjusted and unadjusted odds ratios of different factors affecting the perceived challenge of getting a government job after graduation. Multivariate logistic regression showed that senior students were almost twice (OR = 1.79, *P*=0.001) more likely to perceive getting a government job as a challenge than junior students. The similar patterns were observed in female in comparison with male students (OR = 1.99, *P*=0.001), students with low academic scores in comparison with high academic achievers (OR = 2.14, *P*=0.018), and students with father with low education in comparison with students with father with higher education (OR = 2.02, *P*=0.003).

The analysis of different factors affecting the perceived challenge of getting an admission into specialty training after graduation is presented in Table 4. Senior students were 2.75 times more likely to perceive getting into specialty program as a challenge than junior students (*P*=0.001). Similarly, students whose fathers had low education had a significantly higher likelihood of facing difficulty in securing admission in specialty program (OR = 2.36, *P*=0.001).

After controlling for other variables, senior class year and female gender were associated with perceived challenges of establishing a private dental clinic. Both senior class students (OR = 2.51, *P*=0.001) and female students (OR = 2.02, *P*=0.001) were more likely to perceive the challenges than junior and male students (Table 5).

## 4. Discussion

In Saudi Arabia, most dental colleges have five years of the undergraduate dental program (Bachelor of Dental Surgery, BDS). After successful completion of coursework in five years, students go through one year of internship program after which they can work as a licensed dentist. The employment options for new graduates are either government or private jobs. For the government jobs, the dentists are either employed by the Ministry of Health, Armed Forces hospitals, or as a faculty member in one of the public dental colleges in the country. Some pursue their careers in private dental practice by working as an associate dentist and others open up their own dental clinics. Similarly, a large number of newly graduated dentists apply for higher education/specialty training within the country (Saudi Dental Board) or apply for an admission in advanced training abroad. In addition, few graduates are hired in private dental colleges in the country. Students perceive difficulties that they might face in finding an employment after graduation.

The present study found that most of the dental students perceived potential challenges in their future career. The majority of participants (63.9%) thought that it would be difficult for them to establish a private practice. A previous study conducted in King Saud University, Saudi Arabia, showed that 62% of the samples said that establishing a private dental clinic required a long period of time [14]. It is understandable that new graduates may find it difficult to establish a private clinic because of the challenges of arranging finances, getting approvals from regulatory bodies, facing competition from other dentists, hiring of staff, and networking with dental suppliers. In addition, they lack knowledge of practice management and have no skills or experience of running a private clinic.

On the other hand, the present study also found only 37.6% of the participants perceived difficulty in finding a job in a private dental clinic. Hence, establishing a private clinic was considered a challenge as opposed to finding a job in a private dental setup in Saudi Arabia. The dental students in the country may find it easy to get an employment in private dental practice as opposed to establishing a clinic possibly because of the recent changes imposed by the Ministry of Labor and Ministry of Health that stopped allowing the recruitment and contract renewal of any non-Saudi general dentists [4]. This major change might account for the positive perception of the participants about finding a job in a private dental clinic.

In North America, many dental students choose to work in private practice immediately after graduation instead of perusing postgraduate study in order to pay back their educational debts [15]. According to the American Dental Education Association, the average educational debt of senior dental students was $238,582 in public dental schools and $291,668 in private dental schools in the US. [16]. These debts had a direct effect on students' future career aspirations as it was shown that half of the students planned to work in private practice immediately after graduation because of a large amount of debt they acquire [17]. Canadian dental students revealed that their majority planned to practice general dentistry to pay off their educational debts; however, when the debt was removed as a factor, most students choose to pursue specialty training [18].

In Saudi Arabia, public dental colleges are funded by the government and students do not pay any tuition fee [14]. Moreover, the government provides scholarships to students to pursue higher education from abroad if they fulfill certain requirements. Although Saudi dental students demonstrated awareness about the importance of attaining postgraduate education, 72.4% of them considered it a necessary requirement for a successful career in dentistry [14]. In the present study, about half of the samples found it challenging to pursue postgraduate study abroad or to get acceptance into the Saudi Board programs. This can be explained by the limited number of postgraduate seats offered by the Saudi Commission for Health Specialties as 316 dentists were enrolled in specialty programs in 2013 in the country [5].

A previous study found that 59.3% of Saudi dental students recognized an increase in the number of dental institutions a threat to the dental profession [13]. Therefore, more dentists are entering the dental workforce and they are competing for the limited government jobs or the limited number of seats for specialty programs in the country or abroad. This may explain why more students in our study perceived future career challenges. In the present study, a smaller number of dentists perceived it challenging to find a private job than a government job in the Ministry of Health, Armed Forces, and public dental colleges. The government jobs in Saudi Arabia offer lucrative salary package, opportunities for specialty training/higher education, and retirement benefits. On the other hand, employers of private dental clinics do not offer such competitive salaries and fringe benefits.

In the present study, female students were more likely than male students to perceive the challenges of owning a private clinic (OR = 2.02), getting a government job (OR = 1.91), and pursuing higher education (OR = 1.5). Similarly, previous studies also reported that female students expected more challenges in their career than male students [13, 14, 19]. Females students perceive more challenges because they have to compete with males in professional career in addition to fulfilling family responsibilities and taking care of children [19]. That is why fewer females work full-time and have less average working hours than males [13, 20–22]. More Saudi female dental students considered postgraduate study a necessity for their career than male students [14]. Similarly, female dentists in Bulgaria showed greater interest than males in continuing education [23]. Nevertheless, compared with 77% of male dentists, 54% of female dentists attained postgraduate education because females had family reasons, commitments, and responsibilities in Saudi Arabia [24]. Similar trends were observed in a previous study conducted in New Zealand [20].

High GPA is one of the indicators of employment and job satisfaction by employers and employees [25]. The impact of a high GPA on employment can be explained by two factors: first, high grades are the indicator of productivity, and second, students with higher GPA are more ambitious and efficient in finding jobs [25]. This is in line with what was expressed by students in this study as dental students with high academic scores were twice less likely to perceive career challenges than those with low academic score. In this study, senior students were about twice more likely than junior students to perceive challenges in finding employment and postgraduate qualification. Similarly, a previous study reported that more senior dental students were dissatisfied with dentistry as a profession than junior students [26]. This is probably because senior students are more aware of the employment opportunities and job stresses that lie ahead of them [9]. It has been also suggested that junior students seem to have an idealistic view of the dental career in comparison with senior students [27].

As demonstrated in our study, various influences affect the career challenges of dental students and interns which should be considered while developing career counseling programs. Dental students need career counseling services because they generally do not have enough information about postgraduate educational programs, available employment opportunities, and recruitment and placement processes. For instance, a previous study reported that 62.17% of dental students and interns wanted to pursue education abroad but felt the need for career counseling [28]. Career counseling is also known to significantly influence the specialty choices of postgraduate dental students [29]. Similarly, students perceive guest speaker sessions beneficial because guest speakers provide insight into the practical aspect of the profession by sharing personal examples and career suggestions, and students enjoy real-life learning experience [30]. Likewise, career fairs are strategies that increase the chances of finding a job for students, help them better understand career aspirations, and network with professionals in the field [31]. Therefore, dental colleges should develop career counseling programs, arrange guest speaker presentations, and organize career fairs to ensure optimal preparedness of students for the job market.

The study adds valuable information on the topic of career challenges for dental students and interns, although measures were taken to ensure validity and internal consistency of the self-administered questionnaire to minimize bias in the study. However, one of the limitations of the study is the low response rate. Data collection was achieved by providing an online survey in seven colleges; however, most participants did not respond to the questionnaire. Out of seven dental colleges, only one private college participated in the study. In addition, a cross-sectional study design has inherent limitations of providing a cause-effect relationship between exposure and outcome variables and the direction of associations [32]. In the future, a comparative study about career challenges should be conducted to evaluate the responses of students and faculty from both public and private dental colleges.

## 5. Conclusions

The study found that most dental students perceived challenges of establishing a private dental practice, getting a government job, and securing an admission into specialty program in the country or abroad. Senior students were more likely to perceive future career challenges than junior students. Similarly, female students perceived more difficulties in getting a government job, opening up of a private dental clinic, and obtaining higher education. Low academic score and low father education were associated with an increased likelihood of perceiving future career challenges.

The findings of the study may be utilized by dental institutions to provide courses that prepare students to effectively handle future career challenges. In addition, career counseling may be provided to dental students so that they can have a realistic view of the opportunities available in the job market. This will help them better plan and prepare for their future career aspirations. Dental academia should collaborate with the decision makers in healthcare systems to organize career fairs. Guest speaker presentations (from the government sector, the private sector, and specialty program organizations) may be organized by dental colleges to enrich the awareness of the students about employment situation and avenues for higher education.

## Figures and Tables

**Table 1:** Descriptive analysis: frequency distribution of study variables.

Characteristics	*N* (%)
Gender	
Male	380 (59.7)
Female	257 (40.3)

Academic score in the last year	
Below 80%	154 (24.2)
Between 80 and 90%	243 (38.1)
More than 90%	216 (33.9)

Father's educational level	
No school education	4 (0.6)
School education	143 (22.4)
College education	182 (28.6)
University education	308 (48.4)

Mother's educational level	
No school education	37 (5.8)
School education	182 (28.6)
College education	206 (32.3)
University education	212 (33.3)

Monthly family income	
Less than 7,000 SAR	16 (2.5)
7000–14,000 SAR	89 (14)
15000–20,000 SAR	177 (27.8)
More than 20,000 SAR	255 (40)

**Table 2:** Distribution of students' responses about the challenges after graduation.

Challenges	Agree *N* (%)	Males *N* (%)	Females *N* (%)	*P* value
(1) I think I will have difficulty in finding a job in a private clinic	240 (37.7)	147 (38.7)	93 (36.2)	0.523^*∗*^
(2) I will face competition in getting a job in the Ministry of Health	396 (62.2)	212 (55.8)	184 (71.6)	<0.001
(3) I will have difficulty in getting a job in Armed Forces hospitals	387 (60.8)	215 (56.6)	172 (66.9)	0.009^*∗*^
(4) It will be tough to get the position of a demonstrator/teaching assistant in academia	318 (49.9)	198 (52.1)	120 (46.7)	0.18
(5) It will be very competitive to get an admission into a graduate program aboard	328 (51.5)	169 (44.5)	159 (61.9)	<0.001^*∗*^
(6) It will be hard to get acceptance in Saudi Dental Board	318 (49.9)	178 (46.8)	140 (54.5)	0.059
(7) It will be a challenge to establish your own private dental practice	407 (63.9)	216 (56.8)	191 (74.3)	<0.001^*∗*^

^*∗*^Statistically significant.

**Table 3:** Association between perceived challenges of getting a government job with various factors.

Factors	Unadjusted odds ratio (OR)	*P* value	Adjusted odds ratio (OR)	*P* value
Class year	2.03	0.001^*∗*^	1.791	0.002^*∗*^
Senior students				
Junior students				

Gender	1.99	0.001^*∗*^	1.911	0.001^*∗*^
Female				
Male				

Academic score	1.87	0.018^*∗*^	2.147	0.025^*∗*^
<80%				
≥80%				

Education of father	1.86	0.002^*∗*^	2.024	0.003^*∗*^
Low				
Middle/high				

Education of mother	1.02	0.883	0.917	0.667
Low				
Middle/high				

Family income	0.965	0.875	0.842	0.479
Low				
Middle/high				

^*∗*^Statistically significant.

**Table 4:** Association between perceived challenges of getting an admission in specialty training after graduation with various factors.

Factors	Unadjusted odds ratio (OR)	*P* value	Adjusted odds ratio (OR)	*P* value
Class year				
Senior students	2.46	0.001^*∗*^	2.75	0.001^*∗*^
Junior students				

Gender				
Female	1.36	0.059	1.50	0.038^*∗*^
Male				

Academic score				
<80%	0.51	0.007^*∗*^	0.43	0.005^*∗*^
≥80%				

Education of father				
Low	2.03	0.001^*∗*^	2.36	0.001^*∗*^
Middle/high				

Education of mother				
Low	1.02	0.911	0.95	0.805
Middle/high				

Family income	0.919	0.696	0.76	0.266
Low				
Middle/high				

^*∗*^Statistically significant.

**Table 5:** Association between perceived challenges of establishing a private clinic with various factors.

Factors	Unadjusted odds ratio (OR)	*P* value	Adjusted odds ratio (OR)	*P* value
Class year	2.26	0.001^*∗*^	2.51	0.001^*∗*^
Senior students				
Junior students				

Gender	2.20	0.001^*∗*^	2.02	0.001^*∗*^
Female				
Male				

Academic score	1.22	0.421	1.15	0.648
<80%				
≥80%				

Education of father	1.27	0.234	1.45	0.118
Low				
Middle/high				

Education of mother	1.03	0.852	1.05	0.818
Low				
Middle/high				

Family income	0.822	0.381	0.73	0.207
Low				
Middle/high				

^*∗*^Statistically significant.

## Data Availability

The SPSS data file of this study is available from the corresponding author upon request.
